# Predicting leaf traits of herbaceous species from their spectral characteristics

**DOI:** 10.1002/ece3.932

**Published:** 2014-02-14

**Authors:** Hans D Roelofsen, Peter M van Bodegom, Lammert Kooistra, Jan-Philip M Witte

**Affiliations:** 1 KWR Watercycle Research InstituteNieuwegein, The Netherlands; 2 Department of Ecological science, Subdepartment Systems Ecology, VU UniversityAmsterdam, The Netherlands; 3 Laboratory for Geo-Information Science and Remote Sensing, Wageningen UniversityWageningen, The Netherlands

**Keywords:** Herbaceous species, leaf spectroscopy, leaf traits, small width leaves

## Abstract

Trait predictions from leaf spectral properties are mainly applied to tree species, while herbaceous systems received little attention in this topic. Whether similar trait–spectrum relations can be derived for herbaceous plants that differ strongly in growing strategy and environmental constraints is therefore unknown. We used partial least squares regression to relate key traits to leaf spectra (reflectance, transmittance, and absorbance) for 35 herbaceous species, sampled from a wide range of environmental conditions. Specific Leaf Area and nutrient-related traits (N and P content) were poorly predicted from any spectrum, although N prediction improved when expressed on a per area basis (mg/m^2^ leaf surface) instead of mass basis (mg/g dry matter). Leaf dry matter content was moderately to good correlated with spectra. We explain our results by the range of environmental constraints encountered by herbaceous species; both N and P limitations as well as a range of light and water availabilities occurred. This weakened the relation between the measured response traits and the leaf constituents that are truly responsible for leaf spectral behavior. Indeed, N predictions improve considering solely upper or under canopy species. Therefore, trait predictions in herbaceous systems should focus on traits relating to dry matter content and the true, underlying drivers of spectral properties.

## Introduction

Leaf biochemical and structural properties (better known as leaf traits (Violle et al. 2007)) are indicative for plant strategies (Wright et al. 2004), plant response to pressures (De Bello et al. 2006; Garnier et al. 2007), and ecosystem processes and services (Díaz and Cabido 2001; Lavorel and Garnier 2002; Lavorel et al. 2011). Therefore, ecosystem management and studies are increasingly using traits, for example Douma et al. (2012) and Kokaly et al. (2003). It is recognized that the traits of a leaf influence its spectral properties: reflectance, transmittance, and absorbance (Ustin 2013). Hence, by measuring leaf spectral properties using, for example, spectroscopy (many adjacent spectral bands with high spectral resolution), leaf traits may be approximated (see for a review: (Homolová et al. 2013)). So far, spectroscopic predictions of traits appeared particularly focused on forest ecosystems; for an impressive number of tree species, the spectral properties of individual sunlit top of canopy leaves have been determined using field spectrometers and subsequently related to leaf traits, such as N and P content, photosynthesis rate, leaf mass per area (LMA), water content, lignin, phenolics, tannins, and carotenoids (Asner et al. 2011; Doughty et al. 2011).

For several reasons, tree leaves in the top of the canopy are expected to be similar with respect to growth strategy and nutrient stoichiometry. Firstly, trees reaching the top of a forest canopy have been successful in competing for light, and all have employed trait combinations that maximized growth rates (Falster and Westoby 2005). Moreover, illumination conditions determine nutrient allocation to either light or CO_2_-harvesting compounds, where the former decreases and stabilizes with increasing light exposure (Niinemets 2010). Top of canopy leaves are fully exposed to sunlight and thus spend a proportionally large amount of nutrients to CO_2_ harvesting. This results in a consistent stoichiometry in top of canopy leaves between leaf constituents that codetermine the leaf spectral properties, such as between chlorophyll and N and P (Baraloto et al. 2010). This is acknowledged by, for example, the PROSAIL leaf and canopy radiative transfer model (Jacquemoud et al. 2009), which for modeling canopy reflectance as function of several leaf and canopy properties assumes a fixed proportion of canopy N allocated to canopy chlorophyll (Ustin 2013). Similarly, leaf P can be predicted from spectral data due to its stoichiometric link to leaf N (Asner and Martin 2008b).

What would happen if these prepositions on leaf constituency are no longer valid? Herbaceous ecosystems often contain species with different growth forms and positions mixed across the three-dimensional matrix of the canopy (Aan et al. 2006; Fliervoet and Werger 1984; Hirose and Werger 1995; Kull and Aan 1997). This forces plant species to employ a variety of strategies to acquire sufficient resources. Sufficient light may be collected by investing nutrients in light harvesting compounds (chlorophyll and other pigments), by shifting growth to favorable – unshaded – periods, or by spending nutrients on short-lived fast-growing leaves (Niinemets 2010). Such variety of strategies may affect the coherence of leaf chemical–leaf reflectance relationships, particularly when coinciding with a range of canopy structures (Knyazikhin et al. 2013). These complications potentially disqualify empirical trait–spectra relations that are successfully used in tropical (Asner and Martin 2008b, 2010; Asner et al. 2011; Doughty et al. 2011) and temperate forest (Martin et al. 2008) canopies, for application in herbaceous systems. At the same time, herbaceous ecosystems are widely distributed (Prentice et al. 1992) and of critical importance on climatic processes (Hoffmann et al. 2002). This calls for expanding trait prediction to herbaceous ecosystems.

Therefore, we investigated whether trait prediction is hampered by the variety of plant strategies employed by herbaceous species. We aimed to avoid the potential influence of canopy structure on trait predictions (Knyazikhin et al. 2013; Ustin 2013) and measured traits and spectra therefore directly on leaf level. This research asks whether leaf spectra (here, leaf spectra refer to leaf reflectance, transmittance, and absorbance) remain indicative of key leaf traits when an empirical model – based on partial least squares regression (PLSR) – that correlates spectra to traits is confronted to a wide range of herbaceous species. Despite results on indirectly related traits obtained for forest ecosystems, we hypothesize that only traits that are direct mediators of leaf spectral properties will correlate well with spectral properties.

## Materials & Methods

### Plant collection

We aimed to collect data on traits and leaf spectra from a wide range of ecosystems dominated by herbaceous plant species to encompass the environmental gradients within which herbaceous ecosystems occur (thereby presumably expressing the various strategies viable in herbaceous ecosystems). Thirty-one different plant species were collected in six different ecosystems (dunes, dry and moist heathers, various oligotrophic, and eutrophic grasslands). Four species were sampled in two different ecosystems, resulting in a total of 35 plants. Selection of species was based on an a priori assessment of which species would be abundant and characteristic to each site, as well as aiming to include species from throughout the vertical dimension of the canopy. All plants were sampled during or close to peak growing season (June to August 2011). Specific care was taken for each target species to select a specimen that was a healthy adult with at least six fully developed healthy green leaves that were not affected by herbivores. Whole plants were harvested, if possible, including a portion of the roots to keep the plant as intact as possible. To preserve the plant tissue, the material was wrapped in moist tissues, sealed in a plastic bag, and stored refrigerated until analysis in the laboratory at the end of each day. Fourteen plants were collected during consecutive field work days and were kept refrigerated for max. 48 h.

### Leaf spectra

No more than 30 min after harvesting each plant, leaf spectra (i.e., leaf reflectance, transmittance, and absorbance) were determined for 1–4 (modus = 3) healthy, fully developed leaves, using an ASD (Analytical Spectral Devices, Inc., Boulder, CO) Integrating Sphere (IS) coupled to an ASD FieldSpec Pro FR spectrometer. The IS generates an averaged spectral signature of the leaf that is independent of viewing angle. The FieldSpec collects light with a flexible bundle of optic fibers and transports it to three individual spectrometers that collectively cover the range 350–2500 nm. Preliminary analysis revealed a low signal-to-noise (S/N) ratio for wavelengths >1800 nm as well in the first few spectral bands around 350 nm. To account for this, all spectra were cropped to the 400–1800 nm range, retaining 1401 spectral bands. A second-order Savitzky-Golay filter was applied to remove minor noise. A filter window of 31 nm was applied to 300–800 nm, and a length of 51 nm was applied to the remaining spectral bands. For the remainder of this study, the spectral regions are referred to as follows: visible (VIS, 400–700 nm), near infrared (NIR, 700–1400 nm), and short-wave infrared (SWIR, 1400–1800 nm). In addition to leaf reflectance, transmittance was measured by placing the leaf in front of the IS and recording radiation that penetrated the leaf.

We followed the protocol for measuring reflectance and transmittance as provided by the IS manual (hereafter, standard procedure). Per leaf, a single measurement of reflectance and transmittance (being the average of 100 spectrometer readings) was normalized by dividing the measured radiance by the radiance as reflected by a white reference material (spectralon, Labsphere Inc., North Sutton, NH). This relative measure of reflectance was transformed to absolute reflectance after multiplying by the absolute reflectance of the reference material (which was provided by the manufacturer). Each reflectance and transmittance measurement was corrected for stray light by subtracting radiance measured with a light trap behind the input port. Reflectance and transmittance were calculated per leaf and subsequently averaged for each individual plant. In all, reflectance was acquired for 34 plants, and for 29 plants, noise-free transmittance measurements were available. Overlap between these two groups of plants consisted of 28 plants. For these 28 plants, leaf absorbance was calculated as 1 – reflectance – transmittance. Smoothing of absorbance spectra was not necessary because absorbance was calculated after smoothing was applied to the reflectance and transmittance data.

For three plants, the leaves were too small to cover the IS input port (hereafter: small width leaves), as the IS is designed to receive leaves with a minimum diameter of 10 mm, hereafter: wide leaves. Various solutions have been proposed to measure optical properties of small width leaves (Daughtry et al. 1989; Mesarch et al. 1999; Noda et al. 2013). We applied a correction mechanism developed by Noble and Crowe (2007), which consists of applying a custom-made mask, in our case consisting of a vertical slit 5 mm wide and 10 mm high, reducing the width of the input port and subsequently correcting the measured radiance for the spectral contribution of the mask, based on the masked and unmasked spectra of two reference materials. Small-width leaf transmittance was measured by alternating the leaf background between a white (spectralon) and black reference surface. To assess the reliability of the mask correction, reflectance of all wide leaves was subjected to masked measurements as well. This allowed comparison of leaf reflectance acquired by the standard procedure and spectra resulting from the mask-corrected measurements. Large leaves were not measured for transmittance with the masked protocol due to the labor intensity of this IS setup and time constraints during the fieldwork.

### Leaf traits

Of each plant, the remainder of the plant material was kept refrigerated until the end of the day when they were transported to laboratory facilities. Here, around five leaves (mode value) were selected for plant trait analysis and removed from the plant, excluding petioles. Fresh weight was determined before scanning the leaves on a flatbed scanner to determine leaf area in mm^2^. Samples were oven-dried for 48 h at 60°C to determine dry weight, to thus calculate leaf dry matter content (LDMC, mg/g) as well as the specific leaf area (SLA, mm^2^/mg). After mill grinding the dried samples, leaf nitrogen and leaf carbon contents (LNC, LCC, mg/g) were determined by dry combustion with a Flash EA112 element analyzer (Thermo Scientific, Rodana, Italy). After acid digestion of the ground leaf tissue, leaf phosphorus content (LPC, mg/g) was determined using a color reagent at 880 nm on a spectrophotometer (UV-1601 PC, Shimadzu Corporation, Tokyo, Japan) following the method of Murphy and Riley (1962). In addition, the N:P ratio was calculated to identify the variation in nutrient growth limitations.

Regression between leaf spectra and leaf chemical constituents is influenced by, among other aspects, whether the constituent is expressed on mass or area basis (Grossman et al. 1996). To account for this, we created two new traits by dividing LNC and LPC by SLA, referred to as LNC_area_ and LPC_area_ (g/m^2^). All analyses were carried out for the combined sample of all leaves for each plant, instead of for each leaf individually, to acquire a robust trait value for each plant.

To evaluate the correlation among traits, Pearson's correlation coefficient was calculated between all trait pairs. In addition, a principal component analysis (PCA) was carried out to determine trait variations in multiple dimensions.

### Relation spectra – plant traits

A normal distribution of the trait values was approached after taking a logarithm of the original trait distribution. As an exploratory analysis, Pearson's correlation coefficient between the trait values and each individual spectral band was calculated. Subsequently, plant trait values were related to plant spectra using partial least squares regression (PLSR). The advantage of PLSR over regular multilinear regression is its capacity to deal with colinearity. The 1401 predictor variables (i.e., spectral bands) outnumber the observations and prevent application of regular multivariate regression. PLSR projects the explanatory variables into new orthogonal latent variables (LV, each being a linear combination of the original predictor variables) that explain the variance in the original predictors in an asymptotic fashion. Regression is then applied between the dependant variable (i.e., trait) and an optimal number of LVs (Wold et al. 2001). The number of latent factors for each model was chosen as to minimize the root mean square error (RMSE) of the leave-one-out (LOO) validation. Model accuracy was expressed by RMSE and by the coefficient of determination *r*^2^ which compares predicted to observed values. RMSE and *r*^2^ were acquired during both model calibration (indicated with subscript cal) and after model validation (indicated with subscript val). Correlation coefficients *r* and coefficients of determination *r*^2^ are marked as strong (>0.7), moderate (0.7 ≪ 0.5), or weak (<0.5) (Doughty et al. 2011).

For each of the LOO validation model fittings, the regression coefficients were calculated. A t-test revealed whether the mean regression coefficient deviated significantly from 0. A band was considered significant if *P* < 0.1. PLSR models were iteratively fitted by cropping the predictor variables to the significant predictors of the prior run. This was repeated until all predictors were significant or until cropping did not result in an improved RMSE_val_.

All analyses were performed in R (R_Core_Team 2013) using the pls package (Mevik and Wehrens 2007) and scripts adapted from Feilhauer et al. (2010).

## Results

### Leaf spectra

#### Reflectance

**Figure 1:**
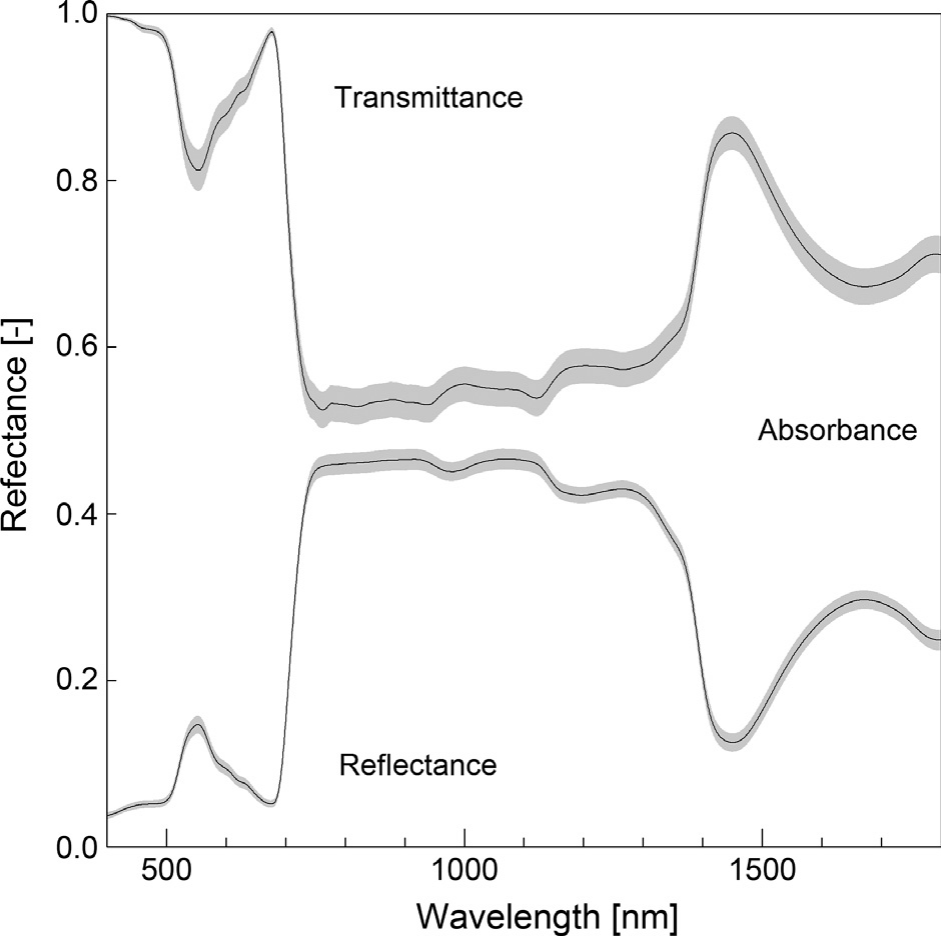
Mean reflectance (*n* = 34) and transmittance (*n* = 29) with the 95% confidence interval indicated in gray, and postsmoothing with a Savitzky–Golay filter. Transmittance is mirrored. The residual of 1 – reflectance – transmittance is defined as absorbance (*n* = 28) and is indicated as such.

Reflectance measurements showed pronounced absorbance in VIS wavelengths and a steep red-edge around 700 nm where variance in reflectance over all plants was very low (Fig. 1). Reflectance values were highest for the NIR region, where 50% reflectance was exceeded. Minor water absorbance features were visible around 1000 and 1200 nm, while major absorbance features were clearly visible around 1450 and 1800 nm. The Savitzky–Golay filter effectively removed spectral noise throughout the spectrum (nonsmoothed spectra not shown).

In addition to reflectance measurements of plants with wide leaves, three plants had small width leaves and were measured with only the masking technique. The correcting algorithms yielded a spectral signature that was typical for a green leaf, however, with increased noise levels in NIR and SWIR compared with unmasked measurements. Many other masked measurements (specifically: masked measurements of wide leaves for validation purposes and a number of additional small width leaves) suffered from abrupt changes in reflectance precisely at the transition between the three spectrometers inside the FieldSpec. These obvious errors are likely due to differences in spectrometer calibration between the moment of sample measurement and measurement of the reference materials. Spectra with abrupt changes were omitted from further analysis, reducing the number of wide leaf plants with dual spectral measurements for validation to just six. Average reflectance and 95% confidence interval of those plants acquired for the unmasked samples and the mask-corrected samples are shown in Supporting Information 1. Noise was considerably higher for masked reflectance, especially around 1000 nm and around 1700 nm. Masked reflectance was lower at the NIR range, but approached the original unmasked reflectance in the remaining parts of the spectrum.

#### Transmittance

**Figure 2:**
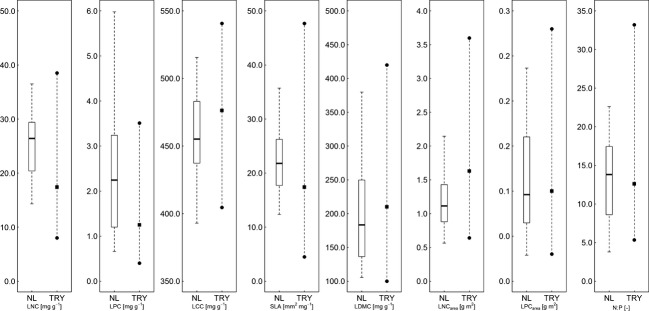
Boxplots of the observed trait values with median and 25% and 75% quantiles (left). To appreciate the range of trait values sampled in this study, the right-hand side shows median and 97.5 and 2.5% quantiles for the same trait derived from the TRY database (Kattge et al. 2011). Note that only summary statistics are provided in Kattge et al. (2011), so it was not possible to plot the exact TRY trait value distribution.

Transmittance spectral signatures followed similar patterns as the reflectance measurements, albeit with considerably more variation in the VIS spectrum (Fig. 2). The NIR range revealed less pronounced absorbance features and several minor peaks, while in the SWIR, a prominent absorbance peak is observed. The Savitzky–Golay filter removed minor noise features around 1000 nm but could not prevent retention of noise in 750–900 nm.

Again, transmittance was measured following the standard procedure where possible. A single plant was measured with the masked procedure, of which in the resulting spectral signatures no oddities were apparent. In the absence of transmittance measurements using both standard and masked procedures, the reliability of the masked transmittance measurements could not be determined.

#### Absorbance

The remainder of emitted radiation that was reflected nor transmitted by the leaf was assumed to be absorbed. Hence, absorbance spectra generally mirrored reflectance and transmittance signatures (Fig. 2). Absorbance in the VIS was consistently high for all plants, with the obvious omission of green spectra (550 nm). Irregular features were observed in the NIR range. Absorbance was the dominant process in the SWIR range around 1450 nm.

### Leaf traits

We observed considerable variation in the values of the seven traits selected to reflect plant strategies concerning nutrient allocation and cycling (Fig. 2). This variation comprises a large part of the global variation in these traits, which is evident from the comparison of the trait values in this study with median and quantile values of the same trait for all records in the TRY database (Kattge et al. 2011): trait values in this study exceeded the 2.5% quantile (LCC, LNC_area_ and LPC_area_) and 97.5% quantile (LNC and LPC) of the TRY database. Other traits values were confined to the range of the TRY database, but covered a large part of the reference trait range. The extremes of the observed trait values correspond with the wide variety in abiotic conditions in the sample locations. SLA values in this study were high compared with the TRY database, reflecting the relatively fertile conditions of the ecosystems included in our study.

Correlation between trait pairs was generally weak (supporting information 2), suggesting that the plants employed various strategies to cope with the environmental conditions. Nutrient-related traits, especially LNC and LPC, correlated weakly with each other as well as to structure-related traits (LCC, SLA, and LDMC) (0.49 > *r* > −0.27), while correlation among structural traits (LCC, SLA, and LDMC) was intermediate (−0.54 > *r* > 0.41). Because LNC_area_ and LPC_area_ are derived from other traits, correlation with the nutrient traits expressed on mass basis was strong (up to *r* = 0.8). In contrast with LPC_area_, LNC_area_ corresponds well also to the structure-related traits. Principal component analysis of the traits (Supporting Information 2, Fig. 2) also suggests low variation among traits, with the first two principal axes accounting for just 68% of the total trait variation. LDMC and SLA on opposite ends of the first axis reveal variation in leaf thickness and area, as expressed as thin large leaves to thick resistant leaves (Wright et al. 2004). N:P ratios in the plants (Fig. 1) indicate that the plants originate from both N (N:P < 14, *n* = 17) and P (N:P > 16, *n* = 11) limited ecosystems, as well as six plants from areas where both nutrients were equally limiting (Koerselman and Meuleman 1996) (mean N:P ratio: 13.3, standard deviation 6.6, Fig. 2).

### Predicting leaf traits from spectra

**Figure 3:**
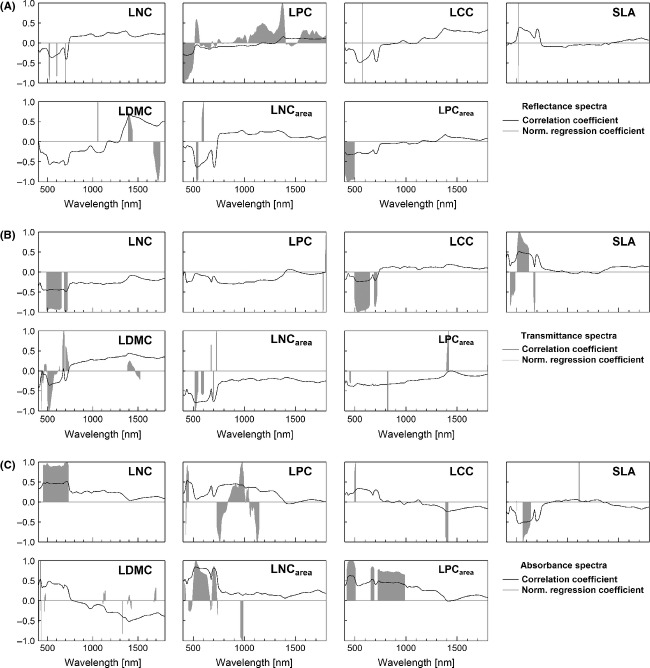
Model summaries for reflectance (A), transmittance (B), and absorbance (C) data. Correlation between each spectral band and traits (solid black line) is highest when approaching 1 (positive correlation) or −1 (negative correlation). Model regression coefficients (dark gray) have been scaled to the maximum and minimum values. Increased deviation from zero signifies additional influence in the model outcome.

**Figure 4:**
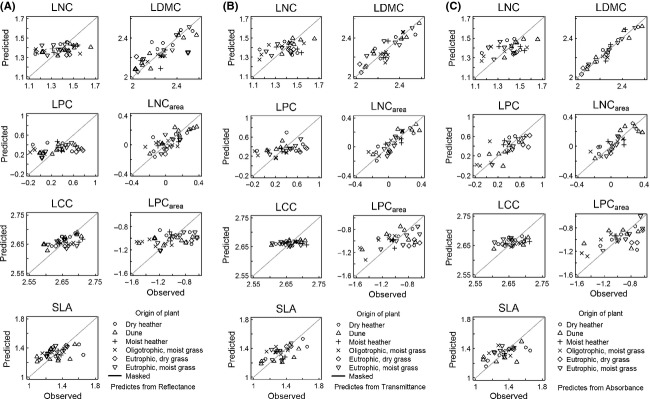
Trait values as observed and predicted from reflectance (A), transmittance (B), and absorbance (C) spectra. Symbols indicate the ecosystem of origin of each plant.

Partial least squares regression was applied to predict plant traits from the different spectra. Prior to this, correlation coefficients between individual spectral bands and traits were calculated to gauge the relation between spectra and traits. Correlation coefficients and normalized model regression coefficients are summarized in Fig. 3A–C, and model performance parameters are provided in Table 1. Scatter plots between observed and predicted trait values are shown in Fig. 4A–C with the ecosystem of origin of each plant indicated. Model residuals were found not to be significantly different between the various ecosystems (one-way ANOVA, *P* < 0.05) except for any LPC model. Likewise, model residuals were not significantly different between small width leaves and plants measured according to the standard procedure.

**Table 1:** Overview of partial least squares regression (PLSR) model performance.

	Reflectance					Transmittance					Absorbance				
	nlv	*r*^2^_cal_	*r*^2^_val_	RMSE_cal_	RMSE_val_	nlv	*r*^2^_cal_	*r*^2^_val_	RMSE_cal_	RMSE_val_	nlv	*r*^2^_cal_	*r*^2^_val_	RMSE_cal_	RMSE_val_
LNC	1	0.10	0.00	0.12	0.13	1	0.21	0.08	0.11	0.12	1	0.24	0.13	0.11	0.12
LPC	2	0.09	−0.22	0.26	0.30	2	0.15	−0.09	0.24	0.27	5	0.54	0.15	0.18	0.25
LCC	2	0.28	0.15	0.03	0.03	1	0.05	−0.08	0.03	0.03	2	0.14	−0.09	0.03	0.03
SLA	2	0.26	0.11	0.12	0.13	2	0.41	0.24	0.11	0.12	2	0.30	0.12	0.12	0.14
LDMC	3	0.67	0.57	0.09	0.10	7	0.78	0.58	0.08	0.12	9	0.93	0.82	0.04	0.06
LNC_area_	2	0.56	0.46	0.10	0.11	3	0.74	0.66	0.08	0.09	2	0.70	0.60	0.09	0.10
LPC_area_	1	0.11	0.00	0.24	0.25	3	0.25	0.05	0.22	0.25	1	0.34	0.21	0.21	0.23

nlv is number of latent variables,% sig is percentage of spectral bands that was significant. *r*^2^ is coefficient of determination for the model calibration and validation (subscript cal and val). Values <0 indicate that model residuals exceed residuals of using mean observation as predictor. RMSE is root mean square error.

#### Structural traits: LCC, SLA, and LDMC

Reflectance spectra were most capable of approaching LCC values (*r*^2^_val_ = 0.15), but still achieved a poor fit. Highest correlation between reflectance and LCC was found in the VIS region, and this was correctly identified by PSLR. SLA prediction accuracy was weak for all spectra, as were the correlations of each band with the SLA values (Fig. 3A–C). The PLSR models identified and employed the highest correlating bands in the VIS, except for the absorbance model. Also, transmittance values of spectral bands around 690 nm that correlated weakly with SLA were incorporated in the PLSR model. LDMC was well predictable with any of the three spectra, but especially with absorbance (*r*^2^_val_ = 0.82, nlv = 9). For transmittance and absorbance, moderately strong correlating bands occurred throughout the VIS and SWIR, while the VIS is almost absent in the reflectance model.

Plotting observed–predicted LDMC values shows only minor deviations from the 1:1 line, while for SLA, strong under and over predictions occurred. Especially, the three highest SLA values are consistently predicted too low, suggesting saturation of the spectral signal at higher SLA values (also found in Asner (1998) and Asner and Martin (2008b)).

#### Nutrient-related traits: LNC and LPC

In contrast to LDMC, nutrient-related traits (LNC and LPC) appeared to be poorly predictable by any spectrum. LNC and LPC were best predicted by transmittance data (*r*^2^_val_ = 0.13 and 0.15, respectively). For some model validations, *r*^2^_val_ was below zero, indicating that the mean observed value is a better predictor than the PLSR model.

For LNC and LPC, bands retained in the band selection procedure coincided with those having the highest correlation coefficients (Fig. 3A–C). LPC – reflectance had no significant bands, so all band were retained. For LPC – absorbance, it is striking that NIR bands correlating positively with LPC are assigned both positive and negative regression coefficients.

Scatter plots of the observed and predicted values for the nutrient-related traits (Fig. 4A–C) reveal a near horizontal point cloud, indicating severe over and under prediction of low and high values, respectively.

#### Nutrient-related traits on area basis: LNC_area_ and LPC_area_

Expressing nutrient content on an area basis nearly always enhanced the correlation with the three spectra, although for LPC_area_, the model accuracy remained weak (*r*^2^_val_ not exceeding 0.21). For LNC_area_ on the other hand, the model accuracy was moderate (and in one instance weak), with *r*^2^_val_ up to 0.66. The four highest LNC_area_ values were structurally under predicted by all spectra (Fig 4A–C), but for the remaining plants, no severe deviations from the 1:1 line were observed.

Correlation between LNC_area_ and transmittance and absorbance spectra was strong in the VIS region. These bands were all identified and employed by the PLSR models. LPC_area_ correlated weakly with reflectance and transmittance spectra and moderately with absorbance in the VIS. For LPC_area_ – absorbance, the PLSR model identified the highest correlating bands and did not incorporate the lesser correlating bands at around 710 nm. Under-and overprediction of extreme values was again a problem for LPC_area_ (Fig 4A–C).

## Discussion

### (first) leaf trait predictions for herbaceous species

This study presents a prediction of leaf traits from leaf spectra for a wide range of herbaceous plant species. While trait prediction of individual leaves from spectral properties receives increased attention (Ustin 2013; Ustin et al. 2009), empirical models have predominantly been developed for trees (e.g., (Asner and Martin 2008b; Asner et al. 2011; Doughty et al. 2011; Martin et al. 2008)). In order to eventually expand trait predictions to currently under-appreciated herbaceous ecosystems, we investigated the feasibility of trait prediction on herbaceous species. To avoid confounding influence from canopy structure (e.g., (Knyazikhin et al. 2013)), we related leaf level spectra directly to leaf level traits. To our knowledge, this is the first study describing leaf level trait predictions of herbaceous species The results found here are relevant for future imaging spectroscopy explorations over herbaceous areas where the spectral signal is not generated by a homogeneous surface of top of canopy leaves, but where canopy gaps and irregularities make that plants with various life forms, light exposure, and strategies contribute to the spectral signal.

In general, the correlation of the seven investigated leaf traits with the various spectra was weak (Table 1). Nutrient-related traits expressed on dry mass content were poorly predictable by any spectrum (reflectance, transmittance, and absorbance), while transmittance and absorbance related strongly and moderately to N content when expressed on an area basis. SLA correlated poorly with all three different spectra. LDMC was reasonably well predicted, especially by absorbance data. Below, we will discuss methodological and ecological reasons for the patterns observed and the implications for future research.

### Methodological issues do not seem to explain low predictive ability

Although unable to successfully model all traits, PLSR model behavior was consistent over all traits and spectra. For all models, the number of latent variables that optimized LOO validation results was relatively low (mode = 2), certainly low compared with other instances of PLSR-predicted vegetation properties, such as Ellenberg indicator values (e.g., (Klaus et al. 2012), mode = 5), plant strategy types ((Schmidtlein et al. 2011), mode = 4), vegetation plot ordination scores ((Feilhauer et al. 2011), mode = 8 & 11), or grassland biomass properties ((Kleinebecker et al. 2012), mode = 8). PLSR models employing a high numbers of latent variables indicate a complex, nonlinear relation between trait and spectra (Haaland and Thomas 1988). Thus, the relatively few latent variables employed here suggest a low information content in the spectral data for our herbaceous species.

The correlation coefficients between each spectral band and the response variable revealed which spectral regions respond to variation in trait value. These regions were generally recognized by the PLSR models and received high absolute regression coefficients, thereby exercising great influence on the model predictions. However, the PLSR models sometimes appear unable to recognize a sudden decrease in correlation between the spectrum and response trait and instead assign high regression coefficients to such bands. A particular prominent decline occurs around 680 nm for nearly all spectrum trait combinations. This decline is likely due to a convergence of spectral response at the onset of the red – edge (∼ 700 nm). Still, some models (e.g., LNC_area_ – absorbance & reflectance) do recognize this decline in correlation and reduce regression coefficients accordingly.

The band selection procedure eliminated bands with low correlation coefficients from the final model (Fig. 3A–C). This reduced the extent to which noise was included in the model and created a convergence between the *r*^2^ of the calibration and validation. On the other hand, bands were sometimes excluded from the model despite a moderately strong correlation with a trait, for example, LDMC – reflectance around 500 and 700 nm. Here, it could have been useful to relax the significance criterion (currently *P* = 0.1) or manually retain bands in the model. Overall, band selection only converged to the highly correlating bands in case of well-correlating traits, for example, LNC_area_ – absorbance, and enhanced these models even further. For other models, band selection could not remedy a poor correlation between the spectrum and traits, for example, LPC – reflectance.

Compared with previous studies on leaf traits–leaf spectra relationships (Asner et al. 2011; Doughty et al. 2011), relatively few plants were investigated here. The power of the PLSR models was thus limited and the low replication contributed to a high RMSE, but did not induce the low *r*^2^ values. Leaves too narrow to cover the integrating sphere sample port, common among plant species found in herbaceous environments, were measured using a mask that reduced the sample port area. The spectral contribution of the mask to the overall signal was removed using reference measurements (Noble and Crowe 2007). While validation of this correction algorithm (Supporting Information 2) proved it to be working reasonably well, this procedure may have introduced additional spectral noise that the PLSR models were unable to resolve, especially in the NIR where differences between the standard procedure and masked measurements were at its largest (Supporting Information 2). Even so, the masked samples did not appear more uncertain in the scatter plots of Fig. 4. Altogether, we think that, although the methodology was not perfect, methodological flaws cannot explain the generally poor ability of spectra to predict leaf traits.

### Physiological perspective of trait predictions

In previous studies relating leaf traits to leaf spectra (Asner and Martin 2008b; Asner et al. 2011; Doughty et al. 2011), correlations between LNC, LPC, LCC, and SLA and reflectance and transmittance exceeded the correlations reported in this research (Table 2).

**Table 2:** Trait prediction accuracy in literature compared with accuracies found here. Indicated are the coefficients of determination (*r*^2^), although in literature, it is not always clear whether this relates to calibration of validation accuracy. Different trait units are indicated on the left-and right-hand side. Spectra used in literature slightly extend beyond the spectral range used in this study (400–1800 nm).

	Asner et al. (2011) RSE		Doughty et al. (2011) Oeco	Asner and Martin (2008a) Front Ecol Environ	Asner and Martin (2008b) RSE		This article		
	R	T	T	R	R	T	R	T	
LNC%	0.77	0.81	0.83	0.55	0.85	0.72	0	0.08	LNC mg/g
LPC%	0.63	0.68		0.47	0.76	0.56	−0.22	−0.09	LCC mg/g
LCC%	0.71	0.74					0.15	−0.08	LPC mg/g
SLA mm^2^/mg				0.79	0.9	0.89	0.11	0.24	SLA mm^2^/mg
LDMC mg/g							0.57	0.58	LDMC mg/g
CWC g/g	0.88	0.9		0.77	0.83	0.87			CWC g/g
LNC_area_ g/m^2^							0.46	0.66	LNC_area_ g/m^2^
LPC_area_ g/m^2^							0	0.05	LPC_area_ g/m^2^

Most notably, LNC proved poorly predictable in this study by any leaf spectrum, although LNC_area_ was predicted with higher accuracy. This improvement is consistent with earlier findings (Grossman et al. 1996), where LNC_area_ prediction also outperformed the prediction of LNC. From the integrating sphere point of view, LNC_area_ is a more direct indication of N content than LNC because the spectrometer receives radiation from a fixed area of the leaf surface. Still, even LNC_area_ was not as well predicted as in previous studies (let alone LNC). This can be attributed to partitioning (i.e., stoichiometry) of leaf N to various N containing leaf constituents that do (e.g., chlorophyll, (Sims and Gamon 2002)) or do not (e.g., CO_2_ fixating molecules, such as rubisco or cell wall material, (Harrison et al. 2009; Hikosaka and Shigeno 2009)) contribute to the leaf spectral signal. Light availability is a strong driver of stoichiometry and other leaf traits (Niinemets 2010; Niinemets and Tenhunen 1997), as well as, to a lesser extent, leaf spectral properties (Lee and Graham 1986; Poorter et al. 1995). Typically, the fraction of total leaf N allocated to chlorophyll and other light harvesting compounds decreases and then stabilizes with increasing illumination of the leaves (Evans and Poorter 2001); when carboxylation instead of light becomes limiting for photosynthesis, leaf N is invested in additional carbon-fixating compounds (Harrison et al. 2009; Niinemets 2010). This suggests that top of canopy leaves, being similarly exposed to illumination levels often above the saturation level for photosynthesis (Poorter et al. 1995), have a consistent fraction of total leaf N allocated to light harvesting compounds (i.e., chlorophyll). Indeed, correlation between leaf chlorophyll and total leaf N can reach up to 50% in temperate forests (Sterner and Elser 2002) and up to 57% in lowland Amazonian forests (Asner and Martin 2010). The herbaceous species investigated here, however, experience a wide range of light availabilities within the complexly structured herbaceous canopy, and as a result, photosynthesis is varyingly limited by light (low light availability, relatively much N allocated to chlorophyll) or carboxylation (high light availability, relatively much N allocated to carboxylating compounds).

While we did not measure chlorophyll content and therefore cannot verify this, we reckon that the leaf N content responsible for driving spectral variation (the chlorophyll fraction) was likely not a fixed proportion of the total leaf N content (the response variable). This is in contrast with the idea that a fixed proportion of canopy N is allocated to chlorophyll, as assumed by the PROSAIL leaf and canopy radiative transfer model (Jacquemoud et al. 2009; Ustin 2013). As such, changes in the spectra by varying chlorophyll content (Poorter et al. 1995) were not mirrored in different LNC nor LNC_area_ values, leaving the PLSR unable to correlate LNC and spectra. This rationale is corroborated when the dataset is partitioned into species exposed to low and high light availability (i.e., lower and upper canopy species, respectively), and LNC and LNC_area_ are again predicted for both groups (supporting information 3). Compared with the original PLSR results (Table 1), LNC was considerably better predicted when only the lower canopy species were taken into account. LNC_area_ performed better with either upper or lower canopy story plants only. These results point to illumination as a driving force on partitioning of leaf N among leaf constituents which in turn influences the relation between spectral properties and total nutrient content.

P lacks an intrinsic spectral signal in the spectral domain commonly used in optical remote sensing (Curran 1989), but is generally correlated with LNC (Mercado et al. 2011), allowing its prediction indirectly through N (Asner and Martin 2008a). In this study, however, LPC was poorly predictable by any of the leaf spectra (Table 2). Our range of N:P ratios was exceptionally large among sites and canopy positions (Fig. 2), imposing different constraints on plant construction and metabolism (Elser et al. 2010) and influencing plant physiology (Güsewell 2004). This implies that even in a small selection of herbaceous species, both N and P are present as limiting nutrient (Koerselman and Meuleman 1996), whereas tropical forest species mainly experience P shortage as soil factor influencing growth rates (Mercado et al. 2011). The nutrient amounts devoted to photosynthetic processes will be dictated by the availability of the limiting nutrient, while the excess of the nonlimiting nutrient is stored in stable leaf compounds that are not involved in photosynthesis nor have a dominant spectral signal. The variety of nutrient limitations may thus explain the poor correlation between spectral data and the total P content.

The weak correlation between LCC and any spectrum could be caused by dominant water absorbance in the SWIR region, which obscures absorbance by, for example, lignin, cellulose, and other carbon-containing leaf compounds (Asner 1998).

Specific leaf area was weakly correlated with reflectance and absorbance (Fig. 3A–C), but relates slightly better to transmittance data. This is ecologically sound, given that SLA is related to leaf density (Niinemets 2001), and transmittance will be modulated by the density of the medium. In literature, the NIR and SWIR ranges were found relevant for SLA predictions for both reflectance and transmittance data (Asner and Martin 2008b). This was not confirmed by findings here, where especially the VIS spectrum proved influential.

We predicted LDMC to align with traits commonly used in ecological applications (Kattge et al. 2011). LDMC is a complementary trait for leaf water content (LWC, mg H_2_O/g dry matter), which is reported as highly correlating with reflectance and transmittance (Asner and Martin 2008b; Doughty et al. 2011). In accordance therewith, LDMC, being complementary to water, was moderately correlated with reflectance and transmittance and strongly to absorbance. The latter correlation was the highest trait–spectrum relation in this study. Water content absorbs radiation at around 1500 nm and 2000 nm, as well as around 1000 and 1250 nm (Asner 1998). However, in our results, mainly spectra around 1400–1450 nm correlated strongly with LDMC, where absorbance dominates both reflectance and transmittance (Fig. 1). In accordance with earlier studies (Asner et al. 2011; Grossman et al. 1996), the spectral signal of leaf water content seems to dominate over contributions of nutrient and dry matter itself. Because of the strong spectral features of leaf water, LDMC is the leaf trait that is best predicted in this study.

Summarizing, Table 2 suggests that trait prediction for herbaceous species does not match findings elsewhere. Only few leaf compounds are directly driving its spectral properties: chlorophyll and other pigments, water and dry matter (Jacquemoud et al. 2009). Using leaf spectra to predict traits that are commonly used for ecological applications, such as LNC and SLA (Kattge et al. 2011), hinges then on a consistent relation with leaf constituents that are the true, underlying, drivers of spectral behavior. Our results suggest that when a variety of growing conditions enforces different environmental constraints (e.g., limitation by either light, N, C, or P), the relation between response traits and spectral relevant traits is less pronounced. Different prevailing plant strategies in herbaceous communities result in an unstable proportional leaf constituency throughout our set of plants which distorts the relation between response traits and the leaf constituents that determine the spectral signal. Consistency in leaf constituents may thus be an additional driver of trait – spectrum relations. This is a new insight into the trait–spectrum relationship in general and in particular for trait modeling in herbaceous systems and suggests that that relations of leaf trait–leaf optical properties may not be easily extrapolated from one ecosystem to another.

### Implications for future research

The poor correlation between traits and leaf spectra, with exception of LDMC and LNC_area_, suggests that trait prediction from imaging spectroscopy in herbaceous ecosystems may be difficult, especially when considering that imaging spectroscopy only records reflectance data (and not transmittance and absorbance) and that the correlation with reflectance exceeded that transmittance and absorbance for LNC and LCC only, while it was still weak. The highest trait–reflectance correlation was for LDMC (highly relevant for wildfire predictions in herbaceous areas (Chuvieco et al. 2010)), encouraging future research to focus on the water and dry matter content of grasslands, as well as traits that are directly responsible for leaf spectra.

However, before claiming far-reaching implications, it should be noted we only took around 30 plants from a small geographic extent. This does not necessarily represent the world's variety in herbaceous flora. Far more herbaceous species from various biomes should be considered to be match the trait and spectral diversity reported in, for example, Doughty et al. (2011). This would firmly establish whether variety of growing conditions and environmental constraints experiences by herbaceous species truly prevent reliable trait prediction for leaf spectral information.

Here, we focused on commonly measured ecological traits, known to be related to (herbaceous) plant strategies, but less so to spectrally relevant leaf properties (Jacquemoud et al. 2009). We expect that for, for example, chlorophyll and pigments, an improved fit may be obtained, as was already demonstrated for tropical leaves (Asner and Martin 2008b). At the same time, for a full exploration and application of such trait predictions, the role of these traits in ecosystems and even more importantly the sources of variation and selection of these traits should be much better understood, for example, to analyze and verify the supposed decoupling between leaf N and chlorophyll content in herbaceous ecosystems. In close collaboration among remote sensing scientists and ecologists, this may be achieved.

## Supporting Information

**Data S1.** Masked and standard procedure reflectance measurements. **Data S2.** Correlation between leaf traits. **Data S3.** LNC and LNC_area_ predictions stratified to canopy position.
