# Supplementary material for: Predicting leaf traits of herbaceous species from their spectral characteristics
Source: Ecol Evol. 2014 Feb 14;4(6):706–19. doi: 10.1002/ece3.932 (PMC3967897; doi:10.1002/ece3.932)
Supplement: Supplementary file 1 — Data S1. Masked and standard procedure reflectance measurements. Data S2. Correlation between leaf traits. Data S3. LNC and LNCarea predictions stratified to canopy position. [file ece30004-0706-sd1.docx]

Supporting information for:

Predicting leaf traits of herbaceous species from their spectral characteristics

H.D. Roelofsen^*A^, P.M. van Bodegom^B^, L. Kooistra^C^ & J.P.M. Witte^A, B^

1. KWR Watercycle Research Institute, Nieuwegein, the Netherlands
2. Department of Ecological science, subdepartment Systems Ecology, VU University, Amsterdam, the Netherlands
3. Laboratory for Geo-Information Science and Remote Sensing, Wageningen University, Wageningen, the Netherlands

* Corresponding author. KWR Watercycle Research Institute, Groningenhaven 7 P.O. Box 1072 3430 BB Nieuwegein, the Netherlands. E-mail: hans.roelofsen@kwrwater.nl.

## 1 Masked and standard procedure reflectance measurements

Reflectance measurements were made using an ASD FieldSpec (FS) Pro FR spectrometer in combination with an integrating sphere (IS). For leaves sufficiently wide to cover the sample port, reflectance was measured following the standard procedure as suggested by the manufacturer. For leaves insufficiently wide to cover the IS sample port, a mask was attached that reduced the sample port width after which the small width leave was exposed to the IS. Correction algorithms ([Noble and Crowe, 2007](#_ENREF_1)) were applied to remove the spectral contribution of the mask and recover the spectral signature of the small width leaf. For six plants, both the standard procedure and the mask-correcting procedure were applied. The accuracy of the mask correction procedure is assessed by comparing standard procedure reflectance measurements and masked-corrected reflectance measurements.


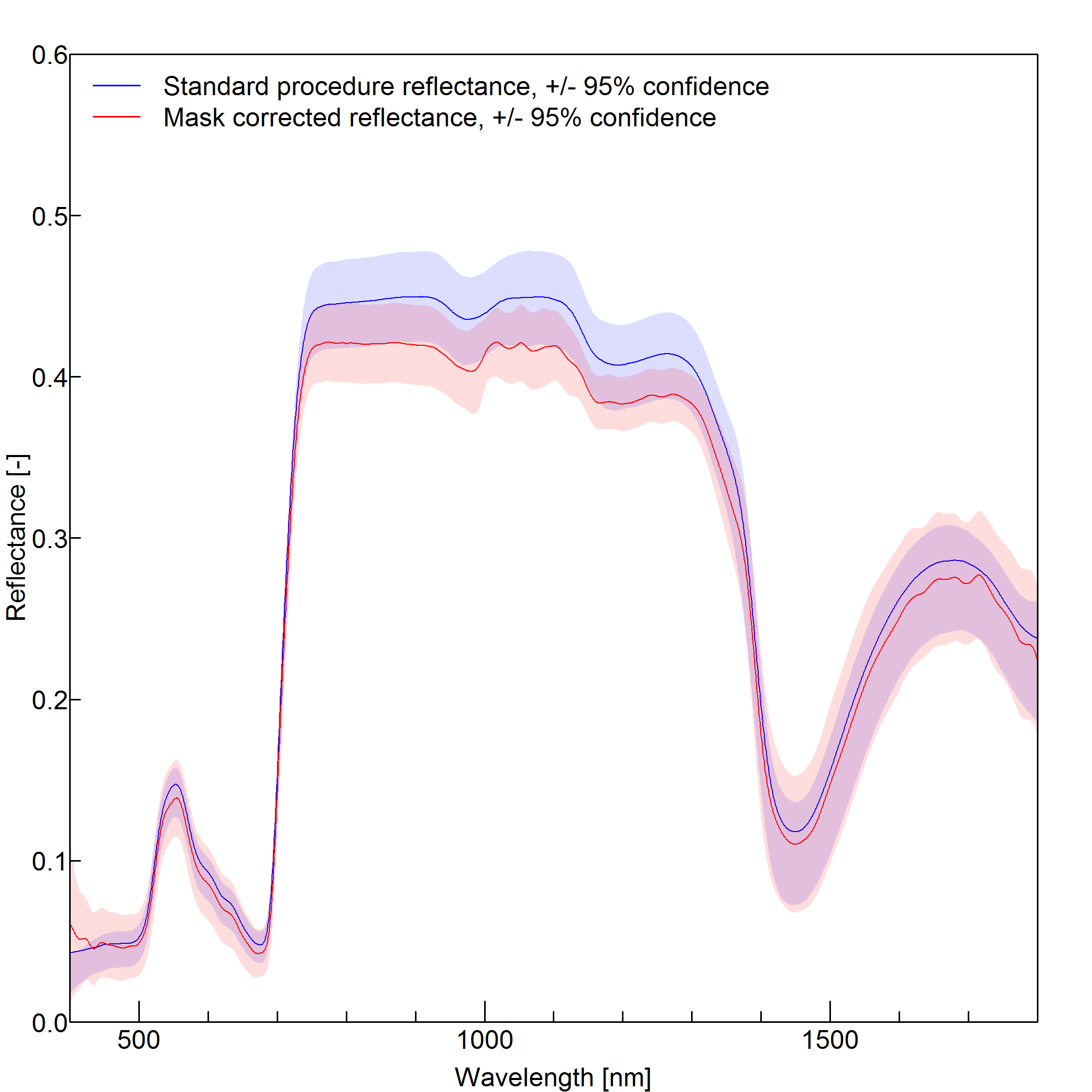


Figure S1 Comparison of reflectance following standard procedure and after applying mask correction procedure.

## 2 Correlation between leaf traits

For 34 plant species, seven plant traits were measured that are supposedly indicative for the plants environment and survival strategy. Correlation among traits was generally low, as is indicated in figure 2. A principal component analysis (PCA) reveals two major axes of variation, where leaf structure (i.e. density and water content as expressed in SLA and LDMC) explains 40% of the total variation and the second axis with nutrient related trait explains an additional 32% (figure 3).


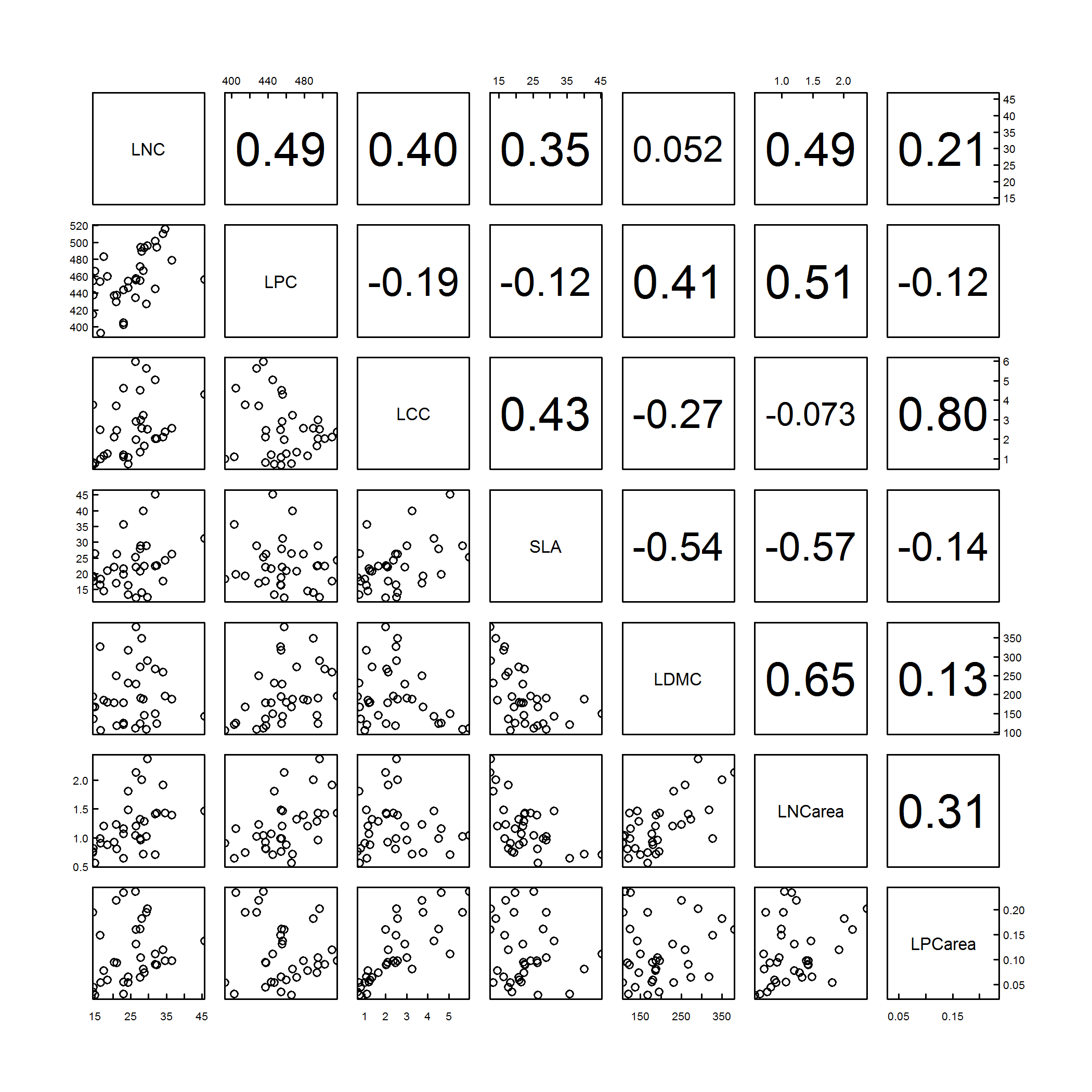


Figure S2 Scatter plots for all trait pairs. Trait values are log corrected, see figure 2 for units.


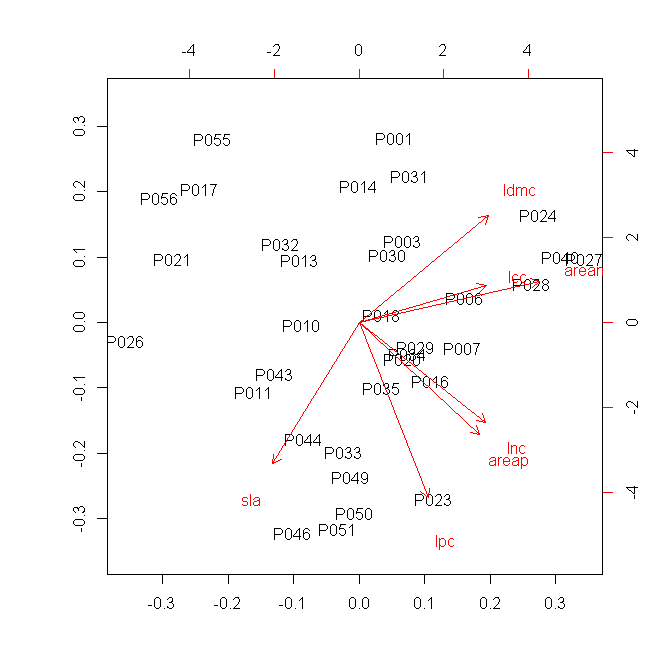


Figure S3 Biplot of principal component analysis of the trait values.

## 3 LNC and LNC_area_ predictions stratified to canopy position

Vertical position in the herbaceous canopy regulates light availability of plant leaves and in turn influences the stoichiometry of nutrients over the various leaf constituents. Shaded plant respond to light limitation for example, by increasing specific leaf area and spending relatively much leaf N in additional light harvesting compounds. Variation in leaf illumination thus drives difference in proportional allocation of leaf N to leaf constituents.

We tested this hypothesis by stratifying the dataset into upper and understory species. Assignment was based on the relative position of the sampled leaves in the canopy; plants clearly surpassing their direct neighbours were labeled upperstory (n= 20, 12 and 11 for reflectance, transmittance and absorbance respectively). In contrast, plant with most leaves (but not necessarily stems and flowers) located close to ground level and below foliage of neighbouring plants were labeled understory (n= 14, 17 and 17 for reflectance, transmittance and absorbance respectively).

We re-ran the PLSR modeling for LNC and LNC_area_ on both subsets (tables 2 and 3). The original model results are included for comparison purposes (table 4). For the understory plants, model performance exceeded the original models. For prediction of LNC for understory species the combined dataset was slightly better. The number of latent variables was high for LNC_area_ ~ reflectance (7 latent variables) and LNC_area_ ~ transmittance (4 latent variables). This suggests high dimensionality information content in the spectral data. While using many latent variables results overoptimistic calibration accuracies, the high adjoining validation accuracy confirms a true high correlation.

Table S1 PLSR modelling results for upper canopy species

Table S2 PLSR modelling results for under canopy species

Table S3 PLSR modelling results for upper and under canopy species combined

## References

NOBLE, S. D. & CROWE, T. G. 2007. Sample holder and methodology for measuring the reflectance and transmittance of narrow-leaf samples. *Applied Optics,* 46**,** 4968-4976.
